# Epidemic history of hepatitis C virus genotypes and subtypes in Portugal

**DOI:** 10.1038/s41598-018-30528-0

**Published:** 2018-08-16

**Authors:** Claudia Palladino, Ifeanyi Jude Ezeonwumelu, Rute Marcelino, Verónica Briz, Inês Moranguinho, Fátima Serejo, José Fernando Velosa, Rui Tato Marinho, Pedro Borrego, Nuno Taveira

**Affiliations:** 1 Research Institute for Medicines (iMed.ULisboa), Faculty of Pharmacy, Universidade de Lisboa, Lisbon, Portugal; 2 Global Health and Tropical Medicine (GHTM), Unit of Medical Microbiology, Instituto de Higiene e Medicina Tropical (IHMT), Universidade Nova de Lisboa, Lisbon, Portugal; 3 Laboratory of Viral Hepatitis, National Center for Microbiology, Institute of Health Carlos III, Majadahonda, Madrid, Spain; 4 Department of Gastroenterology and Hepatology, Santa Maria Hospital, Universidade de Lisboa, Lisbon, Portugal; 5 Centro de Administração e Políticas Públicas (CAPP), Instituto Superior de Ciências Sociais e Políticas, Universidade de Lisboa, Lisbon, Portugal; 6 Centro de Investigação Interdisciplinar Egas Moniz (CiiEM), Instituto Universitário Egas Moniz, Caparica, Portugal

## Abstract

Any successful strategy to prevent and control HCV infection requires an understanding of the epidemic behaviour among the different genotypes. Here, we performed the first characterization of the epidemic history and transmission dynamics of HCV subtypes in Portugal. Direct sequencing of NS5B was performed on 230 direct-acting antiviral drugs (DAA)-treatment naïve patients in Lisbon. Phylogenetic analysis was used for subtyping and transmission cluster identification. Bayesian methods were used to reconstruct the epidemic history of HCV subtypes. Sequences were analysed for resistance-associated substitutions (RAS). The majority of strains were HCV-GT1 (62.6%), GT3 (18.3%, all subtype 3a) and GT4 (16.1%). Among GT1, the most frequent were subtypes 1a (75.5%) and 1b (24.5%). Polyphyletic patterns were found in all but 12 lineages suggesting multiple introductions of the different subtypes in this population. Five distinct epidemics were identified. The first significant HCV epidemic in Portugal occurred between 1930s and 1960s, was caused almost exclusively by GT1b and was likely associated with blood transfusions. Rapid expansion of GT3a occurred in the 1960s and GT1a in the 1980s, associated with intravenous drug use. The most recent epidemics were caused by GT4a and GT4d and seem to be associated with the resurgence of opioid use. The C316N substitution was found in 31.4% of GT1b-patients. Close surveillance of patients bearing this mutation and undergoing dasabuvir-based regimens will be important to determine its impact on treatment outcome.

## Introduction

Hepatitis C virus (HCV) infection continues to be a major public health problem globally despite the introduction of new treatment modalities based on a combination of direct-acting antiviral drugs (DAA). Globally, around 71 million people (1.1% of world population) are chronically infected individuals^1^.

The high genetic heterogeneity exhibited by HCV has given rise to seven major genotypes (1–7)^2^ that differ on average by 30% at nucleotide level^3^, and 86 subtypes^4^ that differ between 15–25% at nucleotide level^3^. It is still unclear if HCV genotypes originated from a single cross-species transmission and subsequently diverged within the human host or from separate zoonotic sources in different regions^5^.

Genotype (GT) 1 accounts for the majority of infections (44%) globally with an extended spatial distribution mainly in upper-middle income and high-income countries^6^. GT3 is the second most prevalent genotype (25%) and is mainly found in lower middle-income countries, mainly in South Asia, and GT4 represents 15% of all HCV infections and is common in resource-constrained countries, mainly in North and Central Africa and the Middle East^6^. There are indications that the different genotypes have existed in restricted geographic areas prior to the twentieth century, GT1 and GT2 in West Africa^7–9^; GT3 in the Indian subcontinent^10^; GT4 in Central Africa^11^; GT6 in Southeast Asia^12^; and GT7 in the Democratic Republic of Congo^13^. The global dissemination of GT1 and GT3 seem to have occurred between 1940 and 1980 through blood transfusion, unsafe medical practices and injecting drug use^14–17^.

The huge genetic variability of HCV brings challenges to host immune control, to the management of HCV-infected patients, and to the development of pan-genotypic treatments^18^. Important differences have been identified in the susceptibility of HCV genotypes to antibody neutralization indicating that a successful vaccination strategy has to take into consideration genotype distribution worldwide^19,20^. Regarding treatment, treatment of chronic HCV infection with peginterferon + ribavirin showed variable efficacy depending on the genotype with sustained virological response (SVR) rates of 42–58%^21–23^, 80%, 66%^24^ or 63–69%^25^ for GT1, GT2, GT3 or GT4, respectively. The current treatment landscape based on a combination of direct-acting antiviral drugs (DAA) has increased dramatically the cure rate of chronic HCV infection^26^. Two drugs targeting the viral NS5B RNA polymerase have been approved in Europe and the US for clinical use, the nucleotide analogue sofosbuvir and the nonnucleoside inhibitor dasabuvir^27,28^, while others are in the pipeline^29^. Sofosbuvir-based combination treatment resulted in SVR rates of 50–93% in six phase 3 trials^30–33^. Since 2014, the combination of sofosbuvir/simeprevir/ribavirin and the new fixed-dose combinations ledipasvir/sofosbuvir and sofosbuvir/velpatasvir resulted in SVR rates of 92–100% in trials enrolling patients infected with HCV GT1^34–40^ and 85–100% in other genotypes^40–43^. Dasabuvir co-packaged with ombitasvir/paritaprevir/ritonavir achieved SVR rates of 89–100%^44^. The presence of resistance-associated substitutions (RAS) as natural polymorphisms in the NS5B gene affects HCV susceptibility to sofosbuvir and dasabuvir and may limit the clinical effectiveness of DAA combinations containing these drugs^45,46^.

Despite the enormous progress made in the treatment of HCV, a recent projection performed in Germany indicates that a substantial number of patients (7%) will fail to achieve SVR and will have limited retreatment options^47^. In addition, people who are cured are at risk of HCV reinfection. Incidence of reinfection varying between 7–13/100 person-years has been reported among gay and bisexual men in Europe^48–50^. Hence, it is necessary to be continuously vigilant about the main factors that may affect the success of HCV treatment in any given location, which are the genotype of the circulating HCV strains and the presence of natural polymorphisms and mutations associated with drug resistance.

In Portugal, there is scarce epidemiological research on HCV and prevalence data are limited. Among the European countries, Portugal has the highest prevalence rate of HCV infection (83.5%) in people who inject drugs (PWID)^51^, while in the prison population, the prevalence of HCV in 2014 was 10.7%^52^. A recent nationwide cross-sectional survey enrolling 1,685 adults in 2012–2014, reported a low HCV prevalence (0.54%; 0.2–0.9)^53^ lower than the previous estimates of 1–1.5%^54,55^. The possible underrepresentation of high-risk groups likely explains the remarkable low HCV prevalence in this recent survey. To comply with the global commitment for hepatitis elimination^56^, at the beginning of 2015, the Portuguese Ministry of Health implemented a national plan of universal access to HCV treatment. In the frame of this programme, named Portal of Hepatitis C, more than 11,700 patients have initiated combinations of sofosbuvir-based treatments and 6,639 patients have been cured, representing 96.5% of those who have already completed the treatment^57^.

Based on four previous studies performed between 1998 and 2014, the majority of HCV infections in Portugal are caused by GT1 followed by GT3, GT4 and GT2^58–63^. The origin, epidemiological history and transmission dynamics of these genotypes in Portugal have not been investigated. Hence, the aims of the present study were to characterize the origin, epidemiological history and transmission dynamics of HCV genotypes and subtypes circulating in Portugal, and to assess the prevalence of natural polymorphisms at the NS5B gene that may impact susceptibility to sofosbuvir and dasabuvir.

## Results

### HCV diversity and transmission dynamics

**Figure 1:**
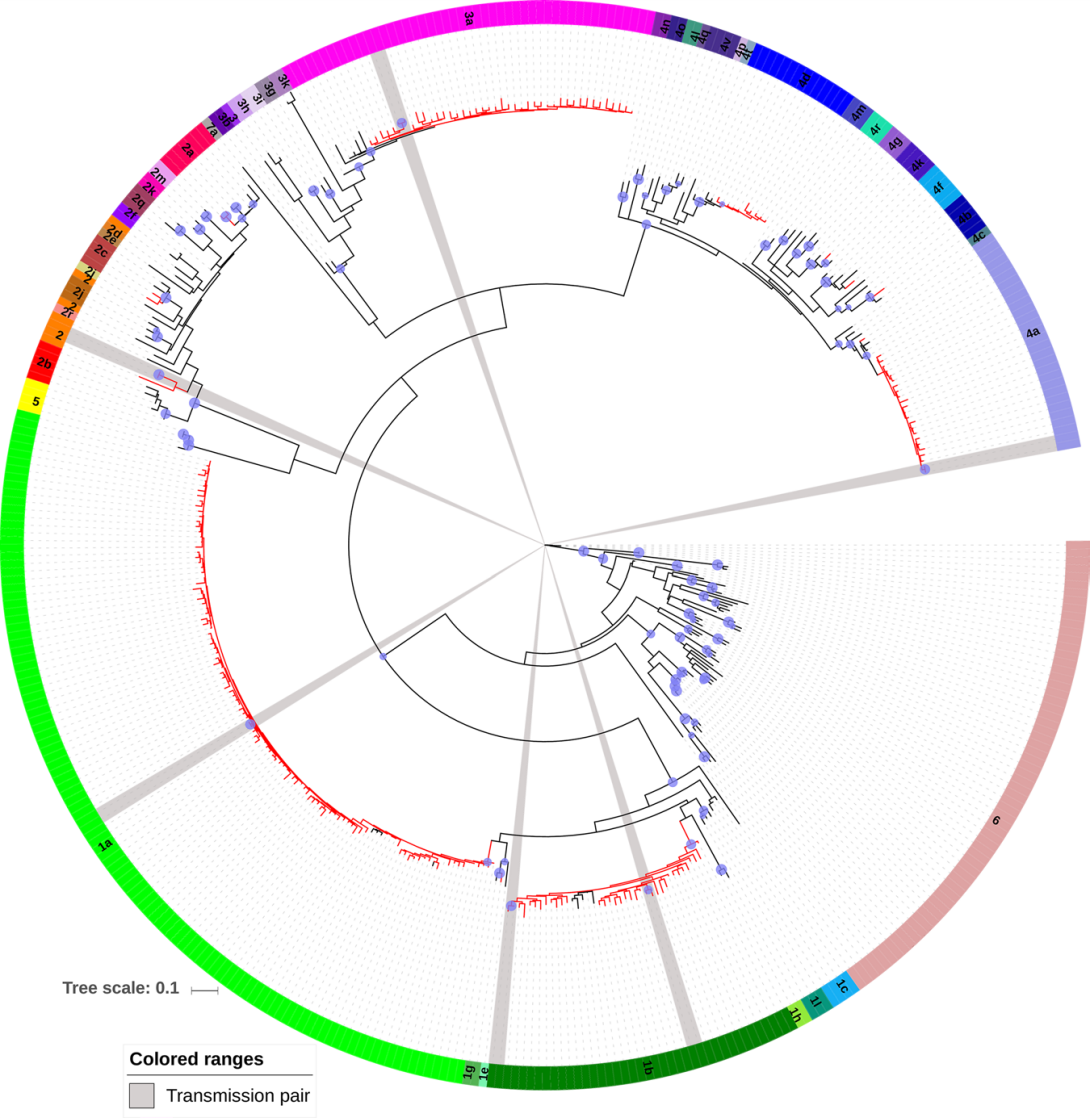
Phylogenetic analysis of NS5B gene sequences from HCV infected patients attending the Hospital Santa Maria, Lisbon, Portugal. The sequences from Portuguese patients are colored in red and reference HCV sequences are colored in black. HCV genotypes and subtypes are indicated with different colored strips with subtype 6 as an outgroup. The bootstrap values supporting the internal branches defining a genotype, subtype or clade are shown at the nodes as blue-filled circles with size corresponding to the magnitude of bootstrap values (only values between 70 and 100% are shown). Bootstrap values of 70% or greater provide reasonable confidence for assignment of an individual sequence to one or the other genotype. The scale represents number of base substitutions per site. Identified transmission clusters (pairs) are shaded in grey.

A total of 230 patients were included in this study. Overall, 59.1% (n = 136) of subjects were men and had a median age of 41 years (IQR: 49–36). The genotyping results showed a majority of GT1 (62.6%; n = 144), followed by GT3 (18.3%; n = 42) and GT4 (16.1%; n = 37), while GT2 was the least frequent (3.0%; n = 7) (Table 1). Among patients harbouring GT1, the most frequent subtype was 1a (75.5%, n = 108) followed by GT1b with a 3.1-fold lower prevalence (24.5%, n = 35), (*P* < 0.0001); only one patient was infected with GT1g (0.4%). Among patients with GT2, 1.3% (n = 3) of the total population had an undetermined subtype, while GT2a and GT2c were each found in 0.9% (n = 2) of the total population. All GT3 were subtype 3a. Among patients with GT4, the most represented subtypes were GT4a, with 10.4% of the total population (n = 24) and GT4d (4.3%; n = 10); GT4b, GT4f and GT4k were found in one (0.4%) patient each (Table 1, Fig. 1).

**Table 1:** Relative frequencies of the HCV genotypes, subtypes and NS5B polymorphisms in patients included in the current study.

HCV genotype/subtype		Age, median (IQR)^#^	Patients with polymorphisms	% of patients with polymorphisms within each genotype/subtype
	N = 230 (%)		N = 216 (93.9%)	%*
1a clade I	23 (10.0)	37 (33–40)	14 (6.5)	60.9
1a clade II	85 (37.0)	40 (36–47)	83 (38.4)	97.6
1b	35 (15.2)	53 (36–60)	32 (14.8)	91.4
1 g	1 (0.4)	53	1 (0.5)	100.0
2	3 (1.3)	57	3 (1.4)	100.0
2a	2 (0.9)	44	2 (0.9)	100.0
2c	2 (0.9)	47	2 (0.9)	100.0
3a	42 (18.3)	42 (35–48)	42 (19.4)	100.0
4a	24 (10.4)	40 (34–46)	24 (11.1)	100.0
4b	1 (0.4)	69	1 (0.5)	100.0
4d	10 (4.3)	42 (39–46)	10 (4.6)	100.0
4 f	1 (0.4)	60	1 (0.5)	100.0
4k	1 (0.4)	46	1 (0.5)	100.0

^#^IQR, interquartile range; *Relative to the total number of patients with polymorphisms (n = 216).

The GT1a clade II was significantly more prevalent than clade I (78.7%; n = 85/108 vs. 21.3%; n = 23/108; *P* < 0.0001). Interestingly, when the GT1a lineages assigned by phylogenetic analysis were analysed with the geno2pheno_[HCV]_ algorithm, 25 sequences had inconsistent clade results (Table S1). Likewise, inconsistent results were observed for two sequences that were classified as GT2 and GT4b by phylogenetic analysis and as GT2b and GT4w by geno2pheno_[HCV]_. Except for 12 HCV lineages which segregated into six transmission clusters (a pair each for GT1a, GT1b, GT2, GT2a, GT3a and GT4a), polyphyletic patterns were found suggesting multiple and old introductions of the different HCV subtypes in this population (Fig. 1).

### Origin and epidemiologic history of HCV strains circulating in Portugal

The dates of the most recent common ancestors (MRCA) of the various HCV subtypes circulating in the Portuguese population were estimated in BEAST under an uncorrelated lognormal relaxed molecular clock with a Bayesian skyline plot (BSP) coalescent demographic model (Table 2). According to our estimates, the ancestor of the 1a strains present in Portugal dated back to 1950 (95% HPD: 1922, 1973) whereas that of 1b dated back to 1946 (95% HPD: 1847, 1965) and 3a to 1963 (95% HPD: 1947, 1977). For genotype 4 strains, similar ancestor dates were obtained [GT4a, 1988 (95% HPD: 1980, 1995); GT4d, 1982 (95% HPD: 1964, 1995)].

**Table 2:** Estimated dates of MRCAs for HCV subtypes identified in the current study.

Dataset	Dates of MRCA^*^ (95% HPD interval)
Subtype 1a (n = 108)	1950 (1922, 1973)
Subtype 1b (n = 35)	1946 (1847, 1976)
Subtype 3a (n = 42)	1963 (1947, 1977)
Subtype 4a (n = 24)	1988 (1980, 1995)
Subtype 4d (n = 24)**	1982 (1964, 1995)

*Mean estimates of most recent common ancestor (MRCA) dates in calendar years.

**Contains additional GT4d sequences (n = 14) from a previous study in Portugal.

**Figure 2:**
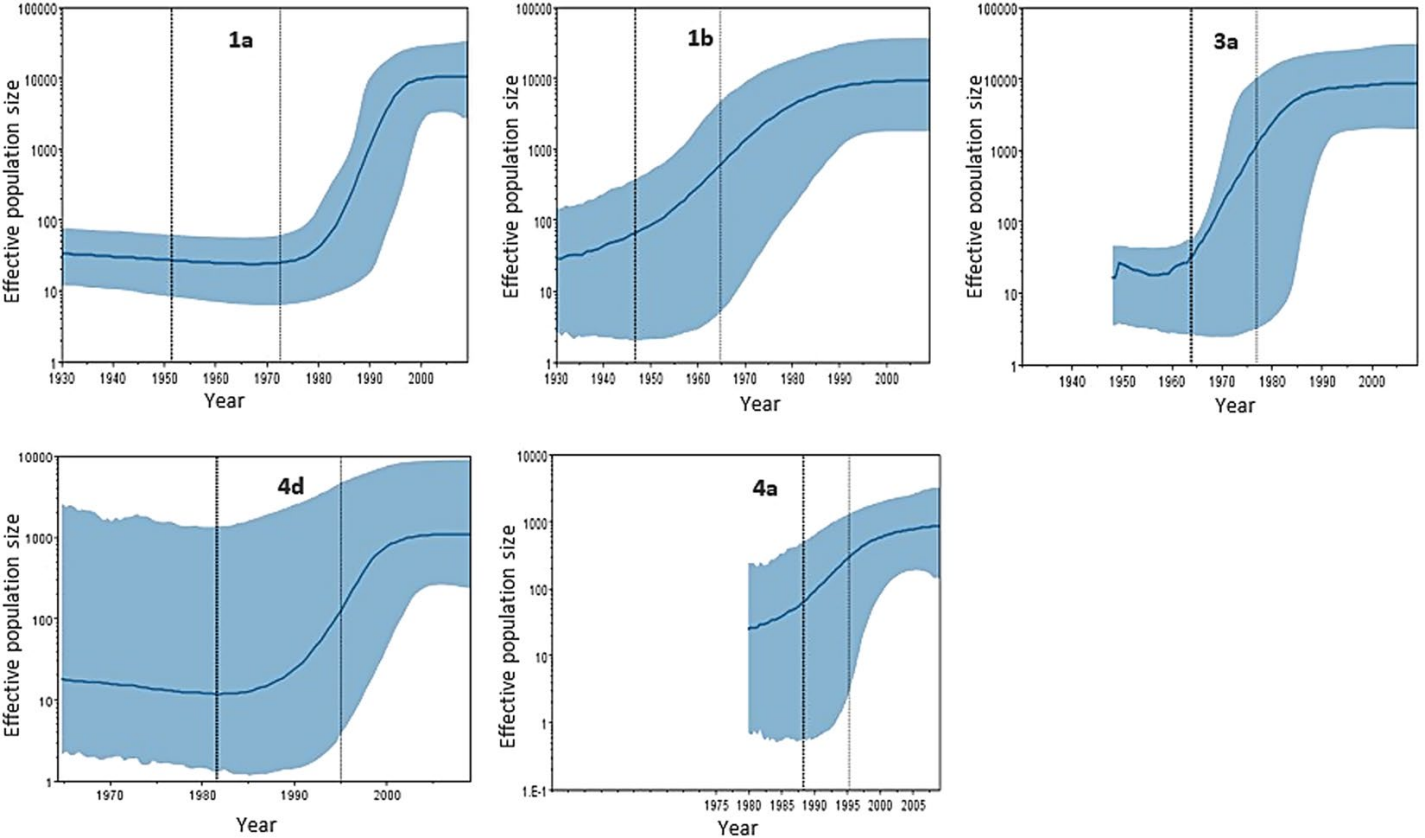
Epidemic history of HCV subtypes in Lisbon, Portugal. Bayesian skyline plot (BSP) showing the epidemic history of the most prevalent HCV subtypes (1a, 1b, 3a, 4a and 4d) found in patients attending the Hospital Santa Maria of Lisbon, Portugal. The solid blue line represents the changes in the mean effective population size through time on a log_10_ scale, with the blue shaded area corresponding to the 95% highest posterior density (95% HPD) interval. The bold dotted and faint dashed black vertical lines represent the median and upper boundaries of the time to the most recent common ancestor (MRCA) respectively. For GT4d, plots were built using additional sequences from Portugal (n = 14) retrieved from GenBank.

To investigate the epidemiologic history of the different HCV subtypes in the Portuguese population, BSP reconstruction was made for each subtype (Fig. 2).

**Figure 3:**
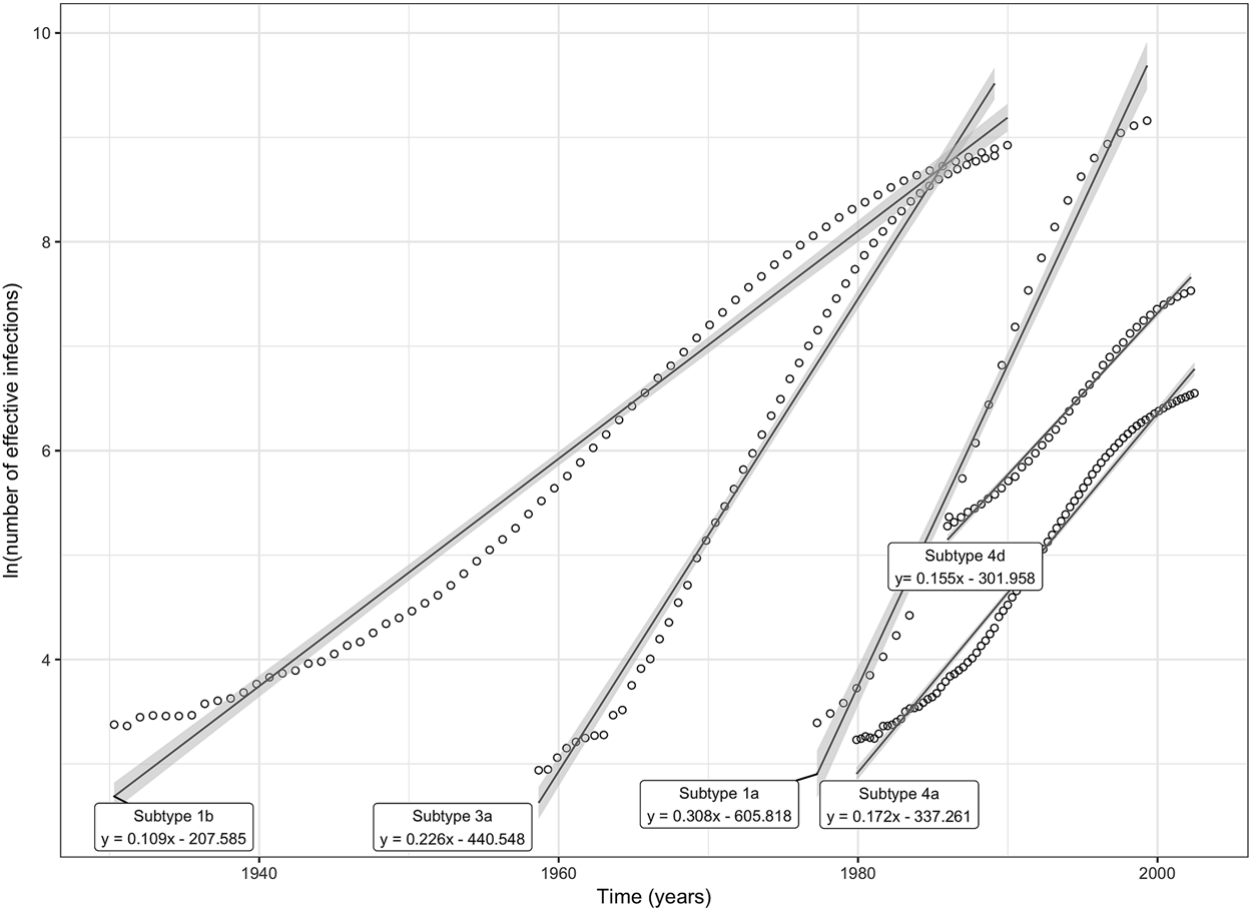
Exponential mean growth rates for the most prevalent HCV subtypes (1a, 1b, 3a, 4a and 4d*) found in the current study. Linear regression equations were derived from the mean growth rates within the exponential phase of the Bayesian skyline plots (BSP) of each subtype as shown in Fig. 2. *Contains additional GT4d sequences (n = 14) from a previous study in Portugal.

The data indicates that the first significant increase in HCV prevalence in Portugal occurred in the first half of the 20^th^ century (the 1930s) and was caused almost exclusively by GT1b until the 1960s. At this time a second epidemic emerged caused by GT3a; a third epidemic caused by GT1a emerged during the 1980s. The first two epidemics caused by GT1b and 3a reached equilibrium at the end of the 1980s, whereas the 1a epidemics continued to grow rapidly (growth rate: 0.308 Ln of number of effective infections per year) until the end of the 1990s when it reached equilibrium. The most recent epidemics were caused by GT4a and GT4d and while the GT4d epidemics seems to have reached an equilibrium GT4a epidemics is still expanding at the present although at a lower rate relative to the epidemic phase of GT1a and GT3a (Fig. 3).

### Baseline polymorphisms and RAS at the NS5B domain

The majority of patients (93.9%; n = 216) harbored viruses with baseline NS5B polymorphisms (Tables 3, 4 and S2). The percentage of patients with polymorphisms within each genotype/subtype was 60.9% for GT1a clade I, 97.6% for GT1a clade II, 91.4% for GT1b and 100% for all the other genotypes/subtypes (Table 1). Within GT1a, more patients with clade II harbored polymorphisms as compared to clade I (97.6%; n = 83/85 vs 60.9%; 14/23; *P* < 0.0001). The only RAS identified was C316N and was observed in 31.4% (n = 11/35) of the patients with GT1b. This amino acid substitution has been shown to confer a 5-fold resistance to the nonnucleoside RdRp palm-1 inhibitor (dasabuvir) in GT1b^64^. In addition, amino acid substitutions on scored position for sofosbuvir, V321I, V321I/V and V321S/L were observed in one patient each harbouring GT1a clade I, GT1a clade II and GT3a respectively (Tables 3, 4 and S2).

**Table 3:** Baseline polymorphisms and amino acid substitutions reported to reduce susceptibility to sofosbuvir and/or dasabuvir in NS5B of HCV strains (GT1 and GT2) found in the current study.

Genotype or subtype														
	1a clade I	N (%)	1a clade II	N (%)	1b	N (%)	1 g	N (%)	2	N (%)	2a	N (%)	2c	N (%)
AA		30		169		68		4		34		10		33
231	S231R	1 (3.3)	S231G/ H/N	14 (8.3)	S231A/N	19 (27.9)							R231S	1 (3.0)
235														
238													S238A	1 (3.0)
241									Q241L	1 (2.9)	R241Q	2 (20.0)	L241Q	1 (3.0)
242	C242S	1 (3.3)											S242C	1 (3.0)
244	D244N	1 (3.3)											S244D	1 (3.0)
245									L245M	1 (2.9)				
246									S246P	3 (8.8)	P246T	2 (20.0)	P246D	1 (3.0)
247									K247E	3 (8.8)			E247P	1 (3.0)
248	Q248K	1 (3.3)											E248Q	1 (3.0)
249									T249A	3 (8.8)				
250											H250R	2 (20.0)		
251	V251I	1 (3.3)					I251V	1 (25.0)					T251V	1 (3.0)
252					A252V	6 (8.8)			V252A	3 (8.8)				
254	K254R	6 (20.0)			R254K	8 (11.8)	R254K	1 (25.0)					H254K	1 (3.0)
258														
262					I262V	5 (7.4)								
266													M266L	1 (3.0)
267											F267L	2 (20.0)		
270			R270K	7 (4.1)									K270R	1 (3.0)
271														
272													Q272E	1 (3.0)
273									A273S	1 (2.9)			S273N	1 (3.0)
276														
282														
285									L285F	3 (8.8)				
289													M289C	1 (3.0)
293									I293L	1 (2.9)				
296														
297													V297I	1 (3.0)
300	R300Q	7 (23.3)	Q300K/ L/R	40 (23.7)	S300A/T	10 (14.7)			S300L	3 (8.8)			K300R	3 (9.1)
303														
304													N304R	1 (3.0)
305														
307							K307G	1 (25.0)						
308									I308V	1 (2.9)			I308L	1 (3.0)
309	Q309R	5 (16.7)	R309Q	23 (13.6)	Q309R	6 (8.8)			I309N	2 (5.9)	I309V	2 (20.0)	V309Q	1 (3.0)
310	D310N	1 (3.3)							K310N	3 (8.8)			A310D	1 (3.0)
311													P311C	1 (3.0)
312														
313														
316					C316N*	11 (16.2)								
321	V321I*	1 (3.3)												
322														
324													S324C	1 (3.0)
327	A327G/ M	3 (10.0)											Q327A	1 (3.0)
328														
329									I329T	3 (8.8)			V329A/ V	2 (6.1)
330	Q330R	1 (3.3)	Q330P	8 (4.7)									E330Q	1 (3.0)
333	A333E	1 (3.3)											E333A	1 (3.0)
334									R334Q	2 (5.9)			R334A	1 (3.0)
335			C335A/ G/S	77 (45.6)	S335N/R	3 (4.4)	S335N	1 (25.0)					N335S	1 (3.0)
336														
337														
338									V338A	1 (2.9)			V338A	1 (3.0)
339														
342														
345														

Notes: Only polymorphic sites whose frequency is above 2% are shown; see Table S3 for the complete dataset of all polymorphisms and mutations.

AA, amino acid position; asterisks indicate resistance associated substitutions (RAS) or substitution on scored position, as detailed below.

V321I and V321I/V, amino acid substitutions on scored position for sofosbuvir (according to data of GT1a).

V321S/L, amino acid substitutions on scored position for sofosbuvir (according to data of GT3).

C316N, RAS: amino acid substitutions reported to reduce the susceptibility of HCV GT1b to nonnucleoside RdRp palm-1 inhibitor (dasabuvir); EC50 (fold change compared to wild-type replicon) is 2–20.

**Table 4:** Baseline polymorphisms and amino acid substitutions reported to reduce susceptibility to sofosbuvir and/or dasabuvir in NS5B of HCV strains (GT3 and GT4) found in the current study.

Genotype or subtype												
	3a	N (%)	4a	N (%)	4b	N (%)	4d	N (%)	4 f	N (%)	4k	N (%)
AA		117		186		9		35		N = 5 (%)		N = 4 (%)
231							K231R	9 (25.7)				
235			V235T	23 (12.4)								
238												
241												
242												
244	N244D	8 (6.8)										
245												
246												
247												
248												
249												
250												
251											K251R	1 (25.0)
252												
254			T254A/ S	15 (8.1)								
258			D258E	22 (11.8)								
262												
266							254			T254A/ S	15 (8.1)	
267			H267R/ Y	4 (2.2)	Y267F	1 (11.1)			H267Y	1 (20.0)		
270							K270R	6 (17.1)				
271												
272	A272D/G/ T	5 (4.3)										
273												
276							I276T/ V	8 (22.9)				
282			T282S	24 (12.9)								
285					Y285F	1 (11.1)	F285Y	3 (8.6)	Y285F	1 (20.0)		
289												
293							L293M	1 (2.9)				
296	Y296F	9 (7.7)										
297					I297L	1 (11.1)						
300							S300N	2 (5.7)				
303												
304	K304R	17 (14.5)	R304K	24 (12.9)	K304R	1 (11.1)						
305												
307	N307G	40 (34.2)	A307G	24 (12.9)			G307R	4 (11.4)				
308												
309							K309R	2 (5.7)				
310					N310D	1 (11.1)						
311					T311Y	1 (11.1)						
312					T312D	1 (11.1)						
313												
316												
321												
322					V322I	1 (11.1)						
324											T324A	1 (25.0)
327	D327G	3 (2.6)										
328											G328S	1 (25.0)
329												
330	D330E/N/ T	13 (11.1)	E330D	9 (4.8)					D330E	1 (20.0)	E330D	1 (25.0)
333	R333G/ K	8 (6.8)			G333K	1 (11.1)						
334												
335												
336			P336S	20 (10.8)								
337	R337G	14 (12.0)										
338												
339												
342									S342G	1 (20.0)		
345			E345Q	21 (11.3)					E345Q	1 (20.0)		

Notes: Only polymorphic sites whose frequency is above 2% are shown; see Table S3 for the complete dataset of all polymorphisms and mutations.

AA, amino acid position; asterisks indicate resistance associated substitutions (RAS) or substitution on scored position, as detailed below.

V321I and V321I/V, amino acid substitutions on scored position for sofosbuvir (according to data of GT1a).

V321S/L, amino acid substitutions on scored position for sofosbuvir (according to data of GT3).

C316N, RAS: amino acid substitutions reported to reduce the susceptibility of HCV GT1b to nonnucleoside RdRp palm-1 inhibitor (dasabuvir); EC50 (fold change compared to wild-type replicon) is 2–20.

## Discussion

We applied phylogenetic and phylodynamic analysis to NS5b sequences obtained from DAA naïve HCV infected patients to make the first characterization of the origin, diversity and epidemiologic history of HCV in Portugal. Like in previous studies, the most prevalent HCV variants were GT1a, representing almost half of the sequences analyzed, followed by GT3a and GT1b^58–62^. These genotypes are responsible for the majority of the HCV cases globally^63,65,66^.

According to our estimates, GT1b has caused a slowly growing but extended epidemic from the 1930s to the 1990s. This epidemic preceded all others by three decades and its features are consistent with widespread unsafe needle practices at the beginning of the 20^th^ century and contaminated blood transfusions which stopped only after the discovery of HCV in 1989 and the subsequent screening of all blood donations for HCV^67,68^. The increase in intravenous drug use in the 1960s has likely sparked the second major HCV epidemic in Portugal which was caused by GT3a and went through the beginning of the 1980s. The fastest growing HCV epidemic in Portugal occurred during the 1980s and 1990s and was caused by GT1a. The GT1a was likely driven by PWID which increased dramatically in the late 1980s and 1990s up to 1% of the Portuguese population due to the rapid social and cultural changes that followed the revolution of 1974 and the increasing availability of heroin^69–71^.

We observed a relatively high (16.1%) prevalence of GT4 among the study population, that is about three-fold higher than the estimated prevalence for Western Europe^65,66^. Similar results were obtained recently in HCV-infected inmates in Portugal (13% GT4)^62^. In addition to GT4a, the most prevalent in our study group, and GT4b and GT4d that have already been reported in Portugal^61,62,72^, we also identified one isolate classified as GT4f and one as GT4k. To the extent of our knowledge, this is the first report of these variants in Portugal. Globally, GT4 has been more prevalent in some countries of Northern and Central Africa and the Middle East^11^, but there are indications that GT4 is becoming increasingly prevalent in Europe, mainly among PWID^61,73,74^.

It was interesting to find that unlike GT1 and GT3 that caused endemic localized infections during decades before becoming generalized, GT4a became epidemic almost immediately after being introduced in Portugal, likely in 1980, and this epidemic may still be growing at a relatively fast rate. The reason for this is unclear as the mode of transmission was known only for 27% of our patients. GT4a dominates the HCV epidemic in Egypt and the main risk factor for its origin and transmission has been unsafe medical practices^75^. Similar to GT4a, the epidemic history of GT4d indicates a recent introduction and a progressive epidemic in the Portuguese population. In Portugal, healthcare related HCV infections are exceedingly rare and GT4a and GT4d have been found in relatively high frequencies (11.3% and 12.1%, respectively) in PWID analysed in a similar period (2008–2009) in Lisbon^61^. In addition, 83.3% of GT4a (n = 6) and 75% of GT4d (n = 4) infected patients for which we have information on transmission risk were PWIDs. We, therefore, believe that injecting drug use is the main mode of transmission of GT4 in Portugal.

The prevalence of GT2 in the study group was only 3.2%, about one-third lower than previously reported data from Western Europe^66^. In particular, the prevalence of GT2a, generally considered as an epidemic subtype, was less than 1%. A similar low prevalence was also observed for GT2c, in contrast to the high frequency (37.5%) previously reported among a limited number (n = 64) of liver biopsies at the Portuguese tertiary Hospital Santa Maria, the same healthcare setting attended by our study group^60^.

Concerning the other less prevalent HCV variants, we identified one individual harbouring GT1g. To the best of our knowledge, this is the first report of HCV-GT1g in Portugal. In Europe, the first partial sequences of GT1g were derived in 1994–1995 from HCV chronically-infected German patients who were probably immigrants from Egypt and Sudan^76^. More recently, the first complete genome of a GT1g isolate derived from a Spanish patient was published^77^.

Recent studies have highlighted distinct geographic distributions of the two clades of GT1a, with clade I being more prevalent in the United States and both clades equally distributed in Europe^78,79^. Furthermore, a recent in-depth phylogenetic analysis has found three distinct sub-clades within clade I^80^. In the present study, that represents the first assessment of clades in Portugal, we found a four-fold higher prevalence of clade II than clade I, and the majority of polymorphisms associate to the former clade. Interestingly, the prevalence of clade I among the patients included in the present study was similar (21%) to that recently observed in DAA-naïve subjects in Spain^81^ but it was significantly lower than in other European countries (e.g. 48% in Italy and 67% in France)^79^. This may indicate a difference in the temporal spread of the HCV-GT1a clades in Portugal as compared to other European countries where both clades are equally distributed.

Baseline NS5B RAS to sofosbuvir has been rarely detected in clinical trial settings^82,83^ and only 0.1% of sequences from all genotypes present in the Los Alamos HCV sequence database harbor RAS to this DAA^84^. Consistent with this, we have not found RAS to sofosbuvir in our patients. On the other hand, the C316N RAS was found in 31% of GT1b-infected patients. This mutation confers low-level resistance to dasabuvir and has been reported in patients failing treatment with sofosbuvir^85,86^. The relatively high prevalence of C316N in our patients is in line with previous reports showing a prevalence of C316N ranging from 11% to 40% in patients with GT1b enrolled in clinical trials, and this mutation was more frequent among European subjects than among those from the United States^87,88^. It should be noted that in the AVIATOR trial that evaluated ritonavir-boosted paritaprevir, ombitasvir and dasabuvir, the C316N RAS at baseline had no significant impact on treatment outcome^87^.

We observed the polymorphism L285F in GT2 which has not been previously reported in the Los Alamos databases as RAS^84^. This mutation was reported in 2017 in a study assessing the genetic heterogeneity of NS5B by ultra-deep pyrosequencing in patients harbouring GT3a not achieving SVR. Interestingly, the L285F polymorphism represented a minor substitution at baseline but was enriched after virological failure^89^. The significance of this mutation is still unclear and needs further investigation. Overall, these data suggest that sofosbuvir and dasabuvir can be used in first-line treatment regimens in Portugal.

Two main limitations should be mentioned. First, our study was based on partial sequences of NS5B which prevented us from examining all RAS in NS5B. Second, the mode of HCV transmission was known only for 27% of the study group preventing us from including this variable in the transmission cluster analysis and from making a more detailed investigation of the risk factors driving the different HCV epidemics in Portugal.

In summary, five distinct epidemics caused by different HCV subtypes were identified over time in Portugal. The first was caused by GT1b, occurred during the 1930s and the 1960s and was likely associated with contaminated blood transfusions. The second and third epidemics were caused by GT3a in the 1960s and GT1a in the 1980s and were likely associated with widespread use of intravenous drug use. The most recent HCV epidemics in Portugal were caused by GT4a and GT4d and seem to be associated with the resurgence of opioid use. The C316N substitution causing low-level resistance to dasabuvir was found in 31.4% of GT1b-infected patients. Close surveillance of patients bearing this mutation and undergoing dasabuvir-based regimens will be important to determine its impact on treatment outcome.

## Methods

### Study Design and Patients

This was a retrospective observational cross-sectional study of consecutive HCV-infected patients seen between November 2007 and July 2009 at the Department of Gastroenterology and Hepatology of the Hospital de Santa Maria in Lisbon which is the main reference center for HCV infection in Portugal. The STROBE checklist was used to help design and conduct the study^90^. Eligibility criteria were: adults (≥18 years of age) who had a diagnosis of HCV infection based on detectable viral load (using the COBAS AMPLICOR HCV MONITOR test, version 2.0 kit of Roche or the Artus® HCV RG RT-PCR Kit of QIAGEN) and were DAA naïve. Stored plasma samples were retrieved from 230 patients (Table S3).

### Ethics

Informed consent was obtained from all subjects. The study was conducted in accordance with the Declaration of Helsinki, as revised in 2013, and was approved by the Institutional Review Board of Santa Maria Hospital (ref. 245/15 of July 30, 2015).

### Amplification and sequencing of HCV NS5B

Viral RNA was extracted from plasma with the QIAmp Viral RNA Mini kit (Qiagen, Hilden, Germany) and a 372 bp region of NS5B gene corresponding to the Okamoto region was amplified using Titan One Tube RT-PCR System (Roche Diagnostics) with the primers HCV_FW1 [5′-CCCGCTGYTTTGACTCVACNGT-3′, location in GT1a isolate H77 (GenBank accession number AF009606), 8264–8285] and HCV_RV1 (5′-CCTRGTCATAGCCTCCGTGAA-3′, location 8636–8616). Amplification was programmed as follows: 2 min at 94 °C and 40 cycles with 1 min at 94 °C; 30 sec at 59 °C and 45 sec (plus 5 sec per min) at 68 °C, and finally, 7 min at 68 °C in the GeneAmp9700 Thermal Cycler. Negative and positive controls were included in all amplification procedures. PCR products were visualized by UV irradiation after electrophoresis on a 2% agarose gel with ethidium bromide. Amplicons were purified with JetQuick PCR Product Purification Spin Kit (GenoMed). Thereafter, the sequencing reaction was performed with the same primers used for amplification and Sanger sequencing was performed (ABI PRISM 3100-Avant Analyser, Applied Biosystems, Foster City, CA) in the following conditions: 1 min at 96 °C and 25 cycles of 10 sec at 96 °C, 5 sec at 56 °C and 4 min at 68 °C (with a sensitivity threshold of approximately 25%)^91^.

### HCV genotype and subtype assignment

This study includes 230 partial sequences of the NS5B region derived from HCV-infected patients. Sequences were 372 nucleotides in length corresponding to positions 8264 to 8636 in reference isolate H77 (GenBank under accession number AF009606)^92^. Table S4 lists the boundaries of the sequences analysed. The nucleotide sequences were aligned with MAFFT algorithm^93^ as implemented in the HIVAlign tool hosted at Los Alamos HIV Database (https://www.hiv.lanl.gov/) and edited using SeaView^94^. HCV subtyping was performed using COMET HCV subtyping tool^95^ and Oxford HCV subtyping tool^96,97^. HCV-GT1a lineages (clade I and clade II) were confirmed by geno2pheno_[HCV]_ (Bonn, Germany; http://hcv.geno2pheno.org/). For HCV subtyping by phylogenetic analysis, nucleotide sequences from reference HCV isolates from each genotype, subtype and clade were retrieved from the HCV Los Alamos Database (https://hcv.lanl.gov/content/index) and International Committee on Taxonomy of Viruses (https://talk.ictvonline.org/ictv_wikis/flaviviridae/w/sg_flavi/56/hcv-classification) and were aligned with the Portuguese sequences using MAFFT. A Maximum likelihood (ML) tree was reconstructed under a GTR + Г nucleotide substitution model and 1000 bootstrap replicates as implemented in raxMLGUI v1.5^98^. The final phylogenetic tree was edited with the graphic resources contained in iTol v4.0.3^99^.

### Transmission cluster analysis

Transmission clusters (TCs: include pairs and clusters ≥3) were identified in the ML tree using Cluster picker v1.2.3^100^ with thresholds for bootstrap supports and genetic distances set at 90% and 4.5% respectively. A sensitivity analysis was also performed to evaluate the influence of bootstrap support (70%, 80% and 90%) and genetic distance thresholds (1.5%, 3.0% and 6.0%) on the clustering^101,102^.

### Bayesian evolutionary analysis: Time-scaled phylogeny

Bayesian phylogenetic trees were reconstructed using the Bayesian Evolutionary Analysis by Sampling Trees software package (BEAST v1.10)^103^ for HCV subtype datasets containing at least 10 dated-sequences spanning the sampling years 2007–2009 (1a, 1b, 3a, 4a and 4d). To improve the evolutionary estimates of GT4d, 14 additional GT4d sequences (sampling years 2008–2009) previously reported in Portugal^61^ were retrieved from the Los Alamos database. A two-codon partitioning model (SRD06 model)^104^, HKY + 4Г nucleotide substitution model, and an uncorrelated lognormal relaxed molecular clock (UCLD) with a Bayesian skyline plot (BSP) coalescent tree prior were selected for the Bayesian analysis. To estimate the date of the most recent common ancestor (MRCA) and the population growth dynamics of the HCV epidemic in Portugal, we specified a Normal prior distribution on the UCLD mean rate parameter (0.001 ± 0.0001 substitutions/site/year)^11,105,106^. For each dataset, three independent MCMC chains were run for 100 million generations with states sampled every 10,000 generations. Log files were combined using Logcombiner to ensure sufficient convergence (ESS ≥ 200) as monitored in Tracer v1.7 (http://tree.bio.ed.ac.uk/software/tracer/) with 10% of posterior samples discarded as burn-in. Maximum clade credibility (MCC) trees were summarized using tree annotator and tree visualization was implemented in Figtree v1.4.3 (http://tree.bio.ed.ac.uk/software/figtree/). To approximate the population growth rate of the epidemic history for each HCV subtype, we performed a linear regression analysis during the exponential growth phase of the BSP curves. The mean exponential growth rates were determined as the slope of the linear regression curves obtained by plotting the mean estimates of the effective population size in the natural logarithmic scale against time (years) in the R software.

### Resistance-associated substitutions (RAS) and polymorphisms

The NS5B gene was reviewed to determine the amino acid substitutions reported to reduce the susceptibility of different HCV genotypes or subtypes to DAA according to the latest review of Pawlotsky 2016, the European and American guidelines^107–110^. Sofosbuvir-specific clinically relevant RAS were L159F, S282T/R, L320I/F/V and V321A, depending on genotype/subtype, and dasabuvir-specific clinically relevant RAS were L314H and C316H/N/Y/W (only for GT1). Other polymorphisms in positions associated with resistance to these drugs were also assessed.

To identify RAS and polymorphisms in NS5B of our patients, the sequences were translated to amino acids and aligned against the following reference sequences: GenBank accession number AF009606 for GT-1a clade I, HQ850279 for GT-1a clade II, EU781827 for GT-1b, AM910652 for GT-1g, KC197237 for GT-2, D00944 for GT-2a, D50409 for GT 2c, D17763 for GT-3a, Y11604 for GT-4a, FJ462435 for GT-4b, DQ418786 for GT-4d, EF589161 for GT-4f, and EU392173 for GT-4k. Degenerate codons were present in some individuals reflecting the presence of quasiespecies. When these codons were present in positions associated with drug resistance all translation possibilities were considered.

### Statistical Analysis

Categorical variables were analysed using the chi-squared test or Fisher’s exact test. *P*-values were 2-tailed and statistical significance was defined as *P* < 0.05. Statistical analyses were performed using the SPSS software Version 22.0 (IBM Corp, Chicago, Armonk, NY, USA).

### GenBank accession numbers

The HCV sequences were deposited in GenBank under accession numbers MG821636-MG821865. The accession numbers for the additional GT4d sequences include: FN401072, FN401080, FN401090, FN401092, FN401095, FN401120, FN401132, FN401142, FN401146, FN401154, FN401157, FN401177, FN401182, FN401193.

## Electronic supplementary material

Supplemetanry TablesS1-S4

Supplementary Data

## Data Availability

Data are available as supplementary information files.
