# Supplementary material for: Epidemic history of hepatitis C virus genotypes and subtypes in Portugal
Source: Sci Rep. 2018 Aug 16;8:12266. doi: 10.1038/s41598-018-30528-0 (PMC6095915; doi:10.1038/s41598-018-30528-0)
Supplement: Supplementary file 1 — Supplemetanry TablesS1-S4 [file 41598_2018_30528_MOESM1_ESM.docx]

**Epidemic history of hepatitis C virus genotypes and subtypes in Portugal**

Authors: Claudia Palladino, Ifeanyi Jude Ezeonwumelu, Rute Marcelino, Verónica Briz, Inês Moranguinho, Fátima Serejo, José Fernando Velosa, Rui Tato Marinho, Pedro Borrego, Nuno Taveira

**Table S1. Sequences with inconsistent clade classification based on the phylogenetic analysis and the geno2pheno_[HCV]_**

| Sequences ID | Clade by phylogenetic analysis | Clade by geno2pheno_[HCV]_ |
| --- | --- | --- |
| 10154.1a.clade2 | 2 | 1 |
| 10509.1a.clade2 | 2 | 1 |
| 10953.1a.clade2 | 2 | 1 |
| 11296.1a.clade2 | 2 | 1 |
| 11519.1a.clade2 | 2 | 1 |
| 11528.1a.clade2 | 2 | 1 |
| 11812.1a.clade2 | 2 | 1 |
| 11869.1a.clade2 | 2 | 1 |
| 11929.1a.clade2 | 2 | 1 |
| 12710.1a.clade2 | 2 | 1 |
| 13096.1a.clade2 | 2 | 1 |
| 13211.1a.clade2 | 2 | 1 |
| 13515.1a.clade2 | 2 | 1 |
| 13602.1a.clade2 | 2 | 1 |
| 13701.1a.clade2 | 2 | 1 |
| 14971.1a.clade2 | 2 | 1 |
| 14973.1a.clade2 | 2 | 1 |
| 15038.1a.clade2 | 2 | 1 |
| 15249.1a.clade2 | 2 | 1 |
| 15637.1a.clade2 | 2 | 1 |
| 15644.1a.clade2 | 2 | 1 |
| 15682.1a.clade2 | 2 | 1 |
| 15710.1a.clade2 | 2 | 1 |
| 15941.1a.clade2 | 2 | 1 |
| 16342.1a.clade2 | 2 | 1 |

**Table S2. Baseline polymorphisms and amino acid substitutions reported to reduce susceptibility to sofosbuvir and/or dasabuvir in NS5B of HCV genotypes and subtypes found in the current study.**

|  |  |  | |  |  | |  |  |  |  |  |  |  |  | **Genotype or subtype** | | |  |  |  |  |  |  | |  |  |  |  |  |
| --- | --- | --- | --- | --- | --- | --- | --- | --- | --- | --- | --- | --- | --- | --- | --- | --- | --- | --- | --- | --- | --- | --- | --- | --- | --- | --- | --- | --- | --- |
|  | **1a clade I** | |  | **1a clade II** | |  | **1b** |  | **1g** |  | **2** |  | **2a** |  | **2c** |  | **3a** |  | **4a** |  | **4b** |  | **4d** |  | | **4f** |  | **4k** |  |
| **AA position** |  | N=30 (%) | |  | N=195 (%) | |  | N=77 (%) |  | N=4 (%) |  | N=34 (%) |  | N=10 (%) |  | N=33 (%) |  | N=131 (%) |  | N=199 (%) |  | N=9 (%) |  | N=35 (%) | |  | N=5 (%) |  | N=4 (%) |
| **231** | S231R | 1 (3.3) | | S231G/H/N | 14 (7.2) | | S231A/N | 19 (24.7) |  |  |  |  |  |  | R231S | 1 (3.0) |  |  |  |  |  |  | K231R | 9 (25.7) | |  |  |  |  |
| **235** |  |  | | T235M | 1 (0.5) | | V235T | 1 (1.3) |  |  |  |  |  |  |  |  | V235T | 1 (0.8) | V235T | 23 (11.6) |  |  |  |  | |  |  |  |  |
| **238** |  |  | |  |  | |  |  |  |  |  |  |  |  | S238A | 1 (3.0) |  |  |  |  |  |  |  |  | |  |  |  |  |
| **241** |  |  | | Q241K | 1 (0.5) | |  |  |  |  | Q241L | 1 (2.9) | R241Q | 2 (20.0) | L241Q | 1 (3.0) |  |  |  |  |  |  |  |  | |  |  |  |  |
| **242** | C242S | 1 (3.3) | | C242S | 2 (1.0) | | C242S | 1 (1.3) |  |  |  |  |  |  | S242C | 1 (3.0) |  |  |  |  |  |  |  |  | |  |  |  |  |
| **244** | D244N | 1 (3.3) | |  |  | |  |  |  |  |  |  |  |  | S244D | 1 (3.0) | N244D | 8 (6.1) | D244N | 1 (0.5) |  |  |  |  | |  |  |  |  |
| **245** |  |  | |  |  | |  |  |  |  | L245M | 1 (2.9) |  |  |  |  |  |  |  |  |  |  |  |  | |  |  |  |  |
| **246** |  |  | |  |  | |  |  |  |  | S246P | 3 (8.8) | P246T | 2 (20.0) | P246D | 1 (3.0) |  |  |  |  |  |  |  |  | |  |  |  |  |
| **247** |  |  | |  |  | |  |  |  |  | K247E | 3 (8.8) |  |  | E247P | 1 (3.0) |  |  | P247A | 1 (0.5) |  |  |  |  | |  |  |  |  |
| **248** | Q248K | 1 (3.3) | | Q248T | 1 (0.5) | |  |  |  |  |  |  |  |  | E248Q | 1 (3.0) |  |  |  |  |  |  |  |  | |  |  |  |  |
| **249** |  |  | |  |  | |  |  |  |  | T249A | 3 (8.8) |  |  |  |  |  |  |  |  |  |  |  |  | |  |  |  |  |
| **250** |  |  | |  |  | | R250K | 1 (1.3) |  |  |  |  | H250R | 2 (20.0) |  |  | R250K | 2 (1.5) |  |  |  |  |  |  | |  |  |  |  |
| **251** | V251I | 1 (3.3) | | V251M | 1 (0.5) | | Q251L | 1 (1.3) | I251V | 1 (25.0) |  |  |  |  | T251V | 1 (3.0) |  |  |  |  |  |  |  |  | |  |  | K251R | 1 (25.0) |
| **252** |  |  | | A252V | 1 (0.5) | | A252V | 6 (7.8) |  |  | V252A | 3 (8.8) |  |  |  |  |  |  |  |  |  |  |  |  | |  |  |  |  |
| **254** | K254R | 6 (20.0) | | K254R | 1 (0.5) | | R254K | 8 (10.4) | R254K | 1 (25.0) |  |  |  |  | H254K | 1 (3.0) |  |  | T254A/S | 15 (7.5) |  |  |  |  | |  |  |  |  |
| **258** |  |  | |  |  | |  |  |  |  |  |  |  |  |  |  |  |  | D258E | 22 (11.1) |  |  |  |  | |  |  |  |  |
| **262** |  |  | |  |  | | I262V | 5 (6.5) |  |  |  |  |  |  |  |  |  |  |  |  |  |  |  |  | |  |  |  |  |
| **266** |  |  | |  |  | |  |  |  |  |  |  |  |  | M266L | 1 (3.0) |  |  |  |  |  |  |  |  | |  |  |  |  |
| **267** |  |  | | T267F | 1 (0.5) | |  |  |  |  |  |  | F267L | 2 (20.0) |  |  |  |  | H267R/Y | 4 (2.0) | Y267F | 1 (11.1) |  |  | | H267Y | 1 (20.0) |  |  |
| **270** |  |  | | R270K | 7 (3.6) | | K270R | 1 (1.3) |  |  |  |  |  |  | K270R | 1 (3.0) | K270R | 1 (0.8) |  |  |  |  | K270R | 6 (17.1) | |  |  |  |  |
| **271** |  |  | |  |  | | G271W | 1 (1.3) |  |  |  |  |  |  |  |  |  |  |  |  |  |  |  |  | |  |  |  |  |
| **272** |  |  | | E272Q | 1 (0.5) | |  |  |  |  |  |  |  |  | Q272E | 1 (3.0) | A272D/G/T | 5 (3.8) |  |  |  |  |  |  | |  |  |  |  |
| **273** |  |  | | N273S | 2 (1.0) | |  |  |  |  | A273S | 1 (2.9) |  |  | S273N | 1 (3.0) | Q273P | 1 (0.8) |  |  |  |  |  |  | |  |  |  |  |
| **276** |  |  | |  |  | |  |  |  |  |  |  |  |  |  |  |  |  |  |  |  |  | I276T/V | 8 (22.9) | |  |  |  |  |
| **282** |  |  | |  |  | |  |  |  |  |  |  |  |  |  |  |  |  | T282S | 24 (12.1) |  |  |  |  | |  |  |  |  |
| **285** |  |  | |  |  | |  |  |  |  | L285F | 3 (8.8) |  |  |  |  |  |  |  |  | Y285F | 1 (11.1) | F285Y | 3 (8.6) | | Y285F | 1 (20.0) |  |  |
| **289** |  |  | |  |  | |  |  |  |  |  |  |  |  | M289C | 1 (3.0) | F289Y | 1 (0.8) |  |  |  |  |  |  | |  |  |  |  |
| **293** |  |  | |  |  | |  |  |  |  | I293L | 1 (2.9) |  |  |  |  | I293L | 1 (0.8) |  |  |  |  | L293M | 1 (2.9) | |  |  |  |  |
| **296** |  |  | |  |  | |  |  |  |  |  |  |  |  |  |  | Y296F | 9 (6.9) |  |  |  |  |  |  | |  |  |  |  |
| **297** |  |  | |  |  | |  |  |  |  |  |  |  |  | V297I | 1 (3.0) |  |  |  |  | I297L | 1 (11.1) |  |  | |  |  |  |  |
| **300** | R300Q | 7 (23.3) | | Q300K/L/R | 40 (20.5) | | S300A/T | 10 (13.0) |  |  | S300L | 3 (8.8) |  |  | K300R | 3 (9.1) |  |  |  |  |  |  | S300N | 2 (5.7) | |  |  |  |  |
| **303** |  |  | | C303S | 1 (0.5) | |  |  |  |  |  |  |  |  |  |  |  |  | I303L/V | 2 (1.0) |  |  |  |  | |  |  |  |  |
| **304** |  |  | |  |  | |  |  |  |  |  |  |  |  | N304R | 1 (3.0) | K304R | 17 (13.0) | R304K | 24 (12.1) | K304R | 1 (11.1) |  |  | |  |  |  |  |
| **305** |  |  | |  |  | |  |  |  |  |  |  |  |  |  |  | A305V | 1 (0.8) |  |  |  |  |  |  | |  |  |  |  |
| **307** |  |  | | G307R | 1 (0.5) | | K307Q | 1 (1.3) | K307G | 1 (25.0) |  |  |  |  |  |  | N307G | 40 (30.5) | A307G | 24 (12.1) |  |  | G307R | 4 (11.4) | |  |  |  |  |
| **308** |  |  | |  |  | |  |  |  |  | I308V | 1 (2.9) |  |  | I308L | 1 (3.0) |  |  |  |  |  |  |  |  | |  |  |  |  |
| **309** | Q309R | 5 (16.7) | | R309Q | 23 (11.8) | | Q309R | 6 (7.8) |  |  | I309N | 2 (5.9) | I309V | 2 (20.0) | V309Q | 1 (3.0) | R309Q | 1 (0.8) | R309K | 2 (1.0) |  |  | K309R | 2 (5.7) | |  |  |  |  |
| **310** | D310N | 1 (3.3) | | D310E | 1 (0.5) | |  |  |  |  | K310N | 3 (8.8) |  |  | A310D | 1 (3.0) | N310D | 1 (0.8) | D310E | 1 (0.5) | N310D | 1 (11.1) |  |  | |  |  |  |  |
| **311** |  |  | |  |  | |  |  |  |  |  |  |  |  | P311C | 1 (3.0) |  |  | C311S | 1 (0.5) | T311Y | 1 (11.1) |  |  | |  |  |  |  |
| **312** |  |  | |  |  | |  |  |  |  |  |  |  |  |  |  |  |  | T312S | 1 (0.5) | T312D | 1 (11.1) |  |  | |  |  |  |  |
| **313** |  |  | | M313L | 1 (0.5) | |  |  |  |  |  |  |  |  |  |  |  |  |  |  |  |  |  |  | |  |  |  |  |
| **316** |  |  | |  |  | | ***C316N**** | 11 (14.3) |  |  |  |  |  |  |  |  |  |  |  |  |  |  |  |  | |  |  |  |  |
| **321** | **V321I*** | 1 (3.3) | | **V321I/V*** | 1 (0.5) | |  |  |  |  |  |  |  |  |  |  | **V321S/L*** | 1 (0.8) |  |  |  |  |  |  | |  |  |  |  |
| **322** |  |  | | V322A | 1 (0.5) | |  |  |  |  |  |  |  |  |  |  |  |  |  |  | V322I | 1 (11.1) |  |  | |  |  |  |  |
| **324** |  |  | |  |  | |  |  |  |  |  |  |  |  | S324C | 1 (3.0) |  |  |  |  |  |  |  |  | |  |  | T324A | 1 (25.0) |
| **327** | A327G/M | 3 (10.0) | | Q327L/R | 3 (1.5) | |  |  |  |  |  |  |  |  | Q327A | 1 (3.0) | D327G | 3 (2.3) |  |  |  |  |  |  | |  |  |  |  |
| **328** |  |  | |  |  | |  |  |  |  |  |  |  |  |  |  |  |  |  |  |  |  |  |  | |  |  | G328S | 1 (25.0) |
| **329** |  |  | | V329I | 2 (1.0) | | T329V | 1 (1.3) |  |  | I329T | 3 (8.8) |  |  | V329A/V | 2 (6.1) | V329I | 1 (0.8) |  |  |  |  |  |  | |  |  |  |  |
| **330** | Q330R | 1 (3.3) | | Q330P | 8 (4.1) | |  |  |  |  |  |  |  |  | E330Q | 1 (3.0) | D330E/N/T | 13 (9.9) | E330D | 9 (4.5) |  |  |  |  | | D330E | 1 (20.0) | E330D | 1 (25.0) |
| **333** | A333E | 1 (3.3) | |  |  | |  |  |  |  |  |  |  |  | E333A | 1 (3.0) | R333G/K | 8 (6.1) |  |  | G333K | 1 (11.1) |  |  | |  |  |  |  |
| **334** |  |  | |  |  | |  |  |  |  | R334Q | 2 (5.9) |  |  | R334A | 1 (3.0) | A334T | 1 (0.8) |  |  |  |  |  |  | |  |  |  |  |
| **335** |  |  | | C335A/G/S | 77 (39.5) | | S335N/R | 3 (3.9) | S335N | 1 (25.0) |  |  |  |  | N335S | 1 (3.0) |  |  | S335G | 1 (0.5) |  |  |  |  | |  |  |  |  |
| **336** |  |  | |  |  | |  |  |  |  |  |  |  |  |  |  |  |  | P336S | 20 (10.1) |  |  |  |  | |  |  |  |  |
| **337** |  |  | |  |  | |  |  |  |  |  |  |  |  |  |  | R337G | 14 (10.7) | P337A | 1 (0.5) |  |  |  |  | |  |  |  |  |
| **338** |  |  | | A338G | 2 (1.0) | | V338A | 1 (1.3) |  |  | V338A | 1 (2.9) |  |  | V338A | 1 (3.0) | A338V | 1 (0.8) | S338N | 1 (0.5) |  |  |  |  | |  |  |  |  |
| **339** |  |  | |  |  | |  |  |  |  |  |  |  |  |  |  |  |  | L339F | 1 (0.5) |  |  |  |  | |  |  |  |  |
| **342** |  |  | | A342V | 1 (0.5) | |  |  |  |  |  |  |  |  |  |  |  |  |  |  |  |  |  |  | | S342G | 1 (20.0) |  |  |
| **345** |  |  | |  |  | |  |  |  |  |  |  |  |  |  |  |  |  | E345Q | 21 (10.6) |  |  |  |  | | E345Q | 1 (20.0) |  |  |

Notes:

AA, amino acids; asterisks indicate resistance-associated substitutions (RASs) or substitution on scored position, as detailed below.

V321I and V321I/V, amino acid substitutions on scored position for sofosbuvir (according to data of GT1a).

V321S/L, amino acid substitutions on scored position for sofosbuvir (according to data of GT3).

C316N, RASs: amino acid substitutions reported to reduce susceptibility of HCV GT1b to nonnucleoside RdRp palm-1 inhibitor (dasabuvir); EC50 (fold change compared to wild type replicon) is 2-20.

**Table S3. HCV subtype and transmission category of the patients included in this study.**

| Subtype | Hetero | Iatrogenic | IDU | Sexual | Transfusion | Total |
| --- | --- | --- | --- | --- | --- | --- |
| 1a |  | 1 | 30 | 1 | 2 | 34 |
| 1b |  |  | 4 |  | 3 | 7 |
| 2a |  |  | 1 |  |  | 1 |
| 2c |  |  |  |  | 1 | 1 |
| 3a | 1 |  | 9 |  | 1 | 11 |
| 4a |  |  | 5 | 1 |  | 6 |
| 4d |  |  | 3 |  |  | 3 |
| Total | 1 | 1 | 52 | 2 | 7 | 63 |

**Table S4. Length and position of the HCV sequences analysed in this study.**

| Region | NT absolute numbering | NT position relative to CDS start in H77 | NT position relative to H77 genome start | AA absolute numbering | AA position relative to CDS start in H77 | AA position relative to polyprotein start in H77 |
| --- | --- | --- | --- | --- | --- | --- |
| NS5B | 1 -> 372 | 664 -> 1035 | 8264 -> 8636 | 1 -> 124 | 222 -> 345 | 2642 -> 2765 |

Notes: Numbering HCV nucleotide sequences was done by analogy to the full length genome sequence of isolate H77 (accession number AF009606).

Abbreviations: AA, amino acid, NA, nucleic acid.
